# A Systematic Review of Occupational Cancers in the Indian Sub-continent

**DOI:** 10.7759/cureus.101061

**Published:** 2026-01-08

**Authors:** Reena Bhardwaj, Neeraj Kumar, Swati Joshi, Ayushi Gupta, Souvik Manna

**Affiliations:** 1 Epidemiology and Biostatistics, Indian Council of Medical Research-National Institute of Child Health and Development Research (ICMR-NICHDR), New Delhi, IND; 2 Epidemiology and Public Health, Indian Council of Medical Research-National Institute of Child Health and Development Research (ICMR-NICHDR), New Delhi, IND; 3 Occupational Health, Indian Council of Medical Research-National Institute of Occupational Health (ICMR-NIOH), Ahmedabad, IND; 4 Medicine, Vardhman Mahavir Medical College and Safdarjung Hospital, New Delhi, IND; 5 Community Medicine, ESIC (Employees' State Insurance Corporation) Medical College and Hospital, Alwar, IND

**Keywords:** carcinogens, exposure assessment, industrial hygiene, occupational cancer, systematic review

## Abstract

Occupational exposure to carcinogens is a major concern in the field of occupational health and public welfare, involving chemicals, metals, dust particles, and organic materials across various industries. This systematic review synthesizes existing literature on occupational cancers in India with respect to the type of carcinogens that the population is exposed to and identifies research gaps. A systematic search was conducted across PubMed, Ovid, Cochrane Library, and other sources, including Google Scholar and manual searches of reference lists, to identify articles published in the English language between January 1, 2000, and October 31, 2024. Eligible studies were those focused on occupational carcinogen exposure in India and available in full text. Two independent reviewers screened the titles and abstracts, followed by full-text evaluation, with any disagreement resolved by a third reviewer. Relevant data were extracted and analyzed thematically. The systematic search yielded 1,360 publications, of which only six studies, comprising a total of 12,360 patients, met the inclusion criteria. These Indian studies highlighted an association between occupational exposure and cancer risk among pesticide workers, farmers, domestic workers, beedi workers, and agricultural laborers, focusing on cancers such as lymphoma, esophageal squamous cell carcinoma (ESCC), lung cancer, cervical carcinoma, buccal mucosa cancer, and gastrointestinal tract cancers. The findings highlight the critical need for conducting further original studies on representative samples of workers exposed to occupational carcinogens to accurately assess the risk of occupational cancers and provide robust evidence on these risks. Furthermore, India should prioritize the systematic monitoring of occupational carcinogens to support hazard control and guide research efforts.

## Introduction and background

Occupational cancer is a significant public health concern in low- and middle-income countries (LMICs) like India, where workers are exposed to various carcinogens at the workplace [1]. 

Occupational exposure to carcinogenic chemicals has long been recognized as a potential source of carcinogenic risk. The term "occupational carcinogen" refers to chemical substances encountered at the workplace, which have been demonstrated or suspected to contribute to tumor development [2]. Despite advances in workplace safety, occupational cancers remain a persistent concern due to ongoing exposure of workers to these carcinogens. The carcinogens are further classified based on the strength of evidence linking them to cancer in humans and animals. The most widely used system is from the International Agency for Research on Cancer (IARC), which divides carcinogens into groups based on the strength of evidence for carcinogenicity. These include Group 1 ("carcinogenic to humans"), Group 2A (“Probably carcinogenic to humans”), Group 2B (“Possibly carcinogenic to humans”), and Group 3 ("Not classifiable as to its carcinogenicity to humans") [3]. Other organizations, like the American Conference of Governmental Industrial Hygienists (ACGIH), use similar categories for confirmed and suspected human carcinogens [4]. 

According to the World Health Organization (WHO) estimates, exposure to occupational and environmental carcinogens is responsible for approximately 19% of cancer diagnoses worldwide, resulting in nearly 1.3 million annual deaths [5]. According to the Global Cancer Observatory (GLOBOCAN) 2022, there were about 20 million new cancer cases globally in 2022 (including non-melanoma skin cancers). The most common new cancer cases in 2022 were lung cancer (12.4%), female breast cancer (11.6%), colorectal cancer (9.6%), prostate cancer (7.3%), and stomach cancer (4.9%) [6]. 

Asbestos, silica, arsenic, and radon are among the most common carcinogens found in the ambient environment. These carcinogens represent a significant category of risk factors that can be mitigated through preventative measures. Mesothelioma shows a very strong association even at lower cumulative exposures to asbestos; risk for lung cancer increases with cumulative exposure and is synergistic with smoking [7]. Risk of silicosis is strongest in workers with higher cumulative exposures to silica; however, disentangling silica’s independent effect from smoking and co-exposures can be challenging [8]. IARC classifies inorganic arsenic as a Group 1 carcinogen, as there is evidence of skin (especially non-melanoma), bladder, lung, and liver and kidney cancers with chronic exposure [9]. Similarly, radon is the second leading cause of lung cancer after smoking and the leading cause among never-smokers [10]. Thus, all four are considered proven human carcinogens (Group 1 and 2A) by IARC [11,12]. Exposures to all such substances remain widespread and are especially intense and uncontrolled in LMICs, including India. As per reports, India was the largest importer of asbestos between 2000 and 2007, and the consumption of asbestos has nearly doubled during this period [13].

The primary occupational diseases of concern in India include silicosis, musculoskeletal disorders (MSDs), coal workers' pneumoconiosis (CWP), byssinosis, chronic obstructive pulmonary disease (COPD), asbestosis, pesticide-related toxicity, and noise-induced hearing impairment (NIHL) [14,15]. In the near future, there will be at least 12.5 million asbestosis patients and 1.25 million asbestos-related cancer patients worldwide, and half of these will be in India [16]. The mean silica dust concentrations in India were found to be 0.47 mg/m^3^, 1.24 mg/m^3^, and 3.28 mg/m^3^ for sandstone, masonry stone, and granite stone mines, respectively [17], which exceeds the proposed limit of respirable crystalline silica (RCS) by the Directorate General of Mine Safety in India (1 mg/m^3^) [18], which in turn is at least three to six times higher than that prescribed by the Occupational Safety and Health Administration (OSHA) [19], American Conference of Governmental Industrial Hygienists (ACGIH) [20], and Egyptian Labor Law standards [21]. Common cancers related to occupational exposure in India include lung, pancreas, bladder, colorectal, breast, and blood cancers. According to a 2022 publication from the Indian Council of Medical Research (ICMR)-backed state registry data, India’s estimated number of new cancer cases in 2022 was 14,61,427 (crude rate: 100.4 per 100,000) [22]. The ICMR also concluded that the incidence of cancer cases is estimated to increase by 12.8% in 2025 as compared to 2020.

Occupational exposure to agents such as respirable crystalline silica dust imposes a heavy “non-cancer” morbidity burden: workers may develop chronic lung diseases, including fibrotic pneumoconioses (e.g., silicosis), chronic bronchitis, emphysema, or airflow-obstructive disease, even in the absence of radiographic silicosis [23]. Silica dust-exposed workers also show increased risk of repeated lung infections, including pulmonary tuberculosis (due to impaired macrophage function) and accelerated decline in pulmonary function over time [24]. Longitudinal cohort data indicate that occupational silica exposure reduces overall survival: in one cohort of diatomaceous-earth workers, silica exposure was associated with a median loss of roughly 0.5 years of life, and significantly greater years lost among those dying of non-malignant respiratory disease [25]. Asbestos exposure causes asbestosis, pleural plaques, diffuse pleural thickening, and chronic respiratory disability long before malignancy develops [26]. Hexavalent chromium, arsenic, and nickel compounds cause chronic dermatitis, neuropathies, hepatic dysfunction, and vascular injury, contributing to non-cancer morbidity in industries such as electroplating and smelting [27]. Diesel exhaust, a Group 1 carcinogen, causes chronic cough, wheeze, asthma exacerbation, reduced lung function, and systemic inflammation mediated by fine particulates and polycyclic aromatic hydrocarbons [28]. Benzene, a hematopoietic carcinogen, produces substantial non-malignant morbidity, including chronic leukopenia, anemia, thrombocytopenia, and immune suppression due to bone-marrow toxicity [29]. Together, these carcinogens lead to chronic respiratory disease, organ damage, disability, and premature mortality independent of their cancer effects.

The etiological factors of lung cancer have become more multifactorial because of increasing industrialization and environmental pollution around the world, as well as in India [30]. Similarly, workers in the textile industry are exposed to various chemicals with known carcinogenic properties, which may increase their risk of developing bladder, colon, and pancreatic cancers [31]. India ranked among the top three countries with the highest attributable tracheal, bronchus, and lung cancer deaths and disability adjusted life-years (DALYs) for occupational exposure to beryllium, cadmium, chromium, diesel exhaust, and polycyclic aromatic hydrocarbons (PAHs), arsenic, silica, and nickel in 2019 [32]. 

The extent of exposure to occupational carcinogens is not well characterized in India. A 2025 scoping review of studies on silicosis in India found that although many localized prevalence studies exist, they come from a small number of states, largely driven by individual investigator interest, and there is “no single paper reporting the overall prevalence in India” [33]. This systematic review aims to address this research gap by synthesizing the scientific evidence and reviewing the studies conducted in India on occupational cancers. The primary objective is to identify the most common occupational cancers in the workplace environment in India. By examining various types of cancers reported in the existing literature, along with associated carcinogens, geographical diversity, observed associations, and research gaps, this review seeks to enhance the understanding of the risk of occupational cancers and support strategies for their mitigation.

## Review

Methodology

This systematic review was conducted following the checklist of items in accordance with the Preferred Reporting Items for Systematic Review and Meta-Analysis Protocol (PRISMA-P) guidelines [34]. It was registered in PROSPERO (International Prospective Register of Systematic Reviews) with registration ID: CRD42025631889.

Literature Search and Selection of Studies

The review included articles published over the past 25 years, from January 2000 to October 2024, using the databases PubMed, Ovid, Cochrane Library, and other sources, including Google Scholar and manual searches of reference lists. The search strategy was comprehensive, using a combination of controlled vocabulary and free-text terms based on the keywords such as “occupational”, “exposure”, “cancer”, “neoplasm”, and “tumor” (Table 1).

**Table 1:** Search strategy used by the authors in current systematic review

Sr. No.	Strategy
1	Occupational exposure* OR Occupat* OR occupant* OR Occupational hazard* OR workplace exposure* OR work place exposure* OR work-related exposure* OR Workplace* OR Work place* OR work hazard* Worker OR Worker* OR job-related exposure* OR job exposure* OR job OR industry* OR agricultur* OR farming* Farmer OR Farmer* OR mining OR construction OR Paint* OR Volunteer* OR Operator* OR Miner*
2	Risk OR associat* OR correlat* OR relation*
3	Neoplasms OR Neoplasm* OR cancer* OR carcinoma* OR tumor* OR tumour* OR malignan*
4	India OR India* OR Andaman OR Nicobar OR Andhra OR Arunachal OR Assam OR Bihar OR Chandigarh OR Chhattisgarh OR “Dadra and Nagar Haveli” OR Daman OR Diu OR Delhi OR Goa OR Gujarat OR Haryana OR Himachal OR Jammu OR Kashmir OR Jharkhand OR Karnataka OR Kerala OR Lakshadweep OR “Madhya Pradesh” OR Maharashtra OR Manipur OR Meghalaya OR Mizoram OR Nagaland OR Orissa OR Odisha OR Pondicherry OR Punjab OR Rajasthan OR Sikkim OR “Tamil Nadu” OR Telangana OR Tripura OR “Uttar Pradesh” OR Uttarakhand OR “Bengal”
5	#1 AND #2 AND #3 AND #4
6	#5 NOT animal

To find the relevant papers, the titles, abstracts, and full-text articles were systematically searched using these terms. Boolean search operations (OR, AND, NOT) and Medical Subject Headings (MeSH) terms were used to combine the above terms to enhance the effectiveness of the search strategy.

Initially, after removing duplicates, two reviewers (RB and AG) independently screened the titles and abstracts of the identified articles to assess their eligibility for inclusion in the review. In cases of disagreement between the reviewers during the multi-step exclusion process, the full-text articles were reviewed and discussed to reach a resolution. If consensus could not be achieved, a third reviewer (NK) was consulted. The full-text articles of all selected references were acquired after the primary screening. The reviewers used Rayyan software (https://www.rayyan.ai/) for the screening of abstracts and titles.

Inclusion and Exclusion Criteria

This review included original full-text studies conducted in India that reported various types of cancers associated with occupational exposures. Studies were considered if they involved human participants exposed to occupational carcinogens and reported results for any type of cancer. This review included retrospective and prospective cohort studies as well as observational and case-control studies in which the population was enumerated based on exposure to known carcinogens. Studies published in the English language between 2000 and 2024 were included.

Studies were excluded if they did not provide sufficient details about exposure assessment or relied solely on self-reported assessments. Grey literature was excluded as the authors aimed for a rapid review and did not include meta-analysis, requiring very high methodological comparability. Abstracts, letters, case reports, conference proceedings, comments, editorials, and methodology papers were not considered. Previously published risk analyses, such as meta-analyses and systematic reviews, were also excluded. Articles not accessible in full text and published in languages other than English were not included. Additionally, studies focusing on endpoints other than cancer, such as biomarkers, genetic analyses, or exposure assessments without outcome ascertainment, were excluded from this review.

Data Extraction

The data extraction was conducted independently by two reviewers using a structured data extraction form (Table 2).

**Table 2:** Information collected in the data extraction form

Sr. No.	Information Variables
1	Lead Author
2	Title of publication
3	Year of publication
4	Design of study
5	Setting of research (State)
6	Population under study
7	Number of participants in the study (both groups)
8	Occupation of participants
9	Type of cancer reported
10	Findings from the study
11	Conclusions

This form collected the detailed information from each included study, including the name of the first author, date of publication, study title, study location, study design, sample size, duration of exposure, type of occupation, type of cancer reported, and key outcomes such as odds ratios (OR) with their 95% confidence intervals (CIs). In cases where information or study procedures were incomplete or unclear, a single attempt was made to contact the corresponding author for clarification. If specific data for any outcome were not reported, it was recorded as missing, and no imputation or assumptions were made for the unavailable data. This systematic and transparent approach ensured consistency and accuracy in the data extraction process.

Results

**Figure 1:**
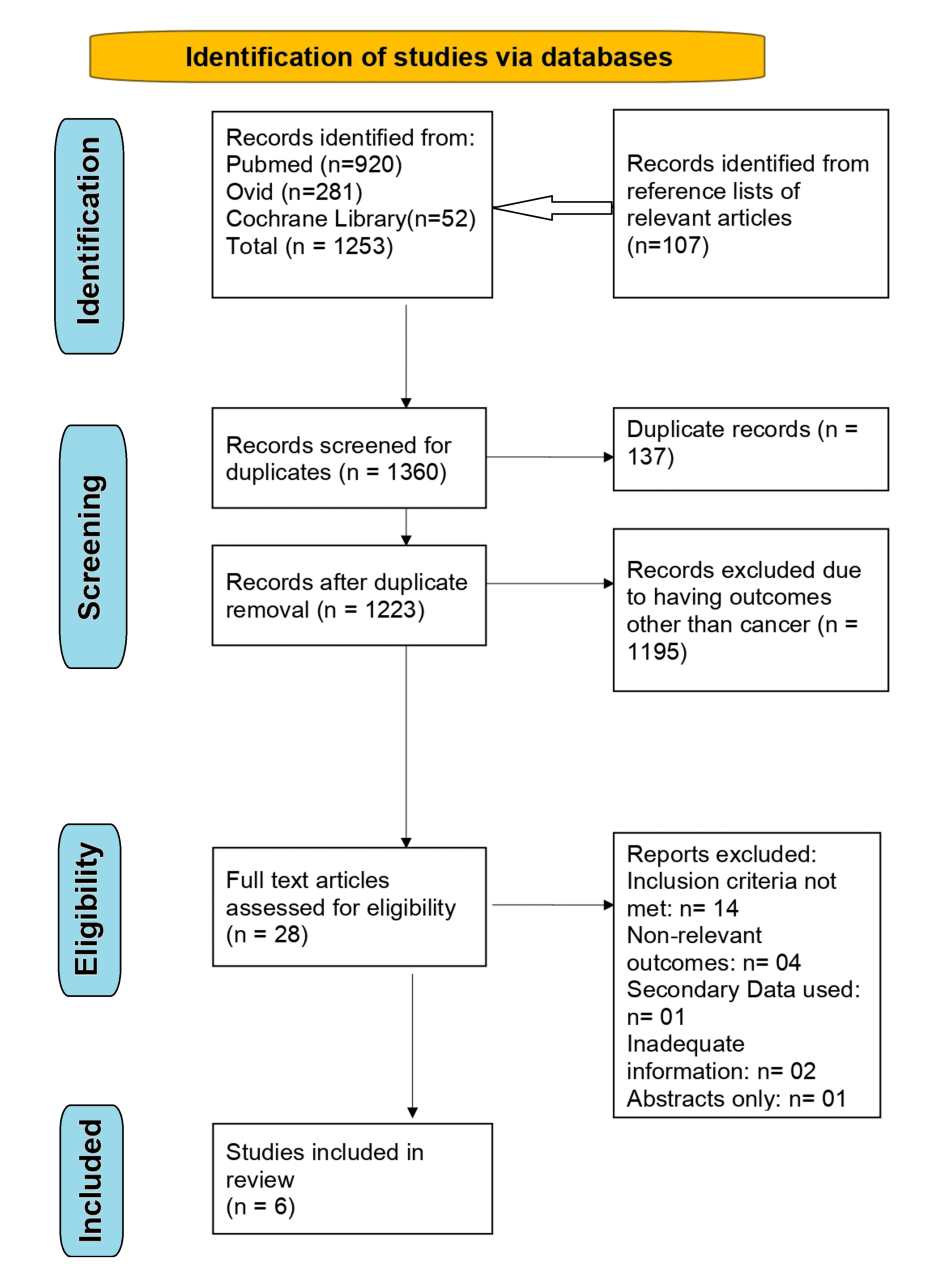
PRISMA flow chart PRISMA: Preferred Reporting Items for Systematic reviews and Meta-Analysis

The PRISMA flow diagram outlining the study selection process is shown in Figure 1.

The initial systematic search identified a total of 1,360 publications, comprising 920 from PubMed, 281 from Ovid, 52 from Cochrane Library, and 107 from Google Scholar and manual searches of reference lists. After removing duplicates (n=137), 1,223 publications remained for screening. Following the title and abstract screening, 1195 studies were excluded as they evaluated occupational exposures in relation to health outcomes other than cancer. The remaining 28 full-text articles were evaluated for eligibility, of which 22 papers were excluded for not meeting the inclusion criteria or for reporting unrelated outcomes. Ultimately, six studies were included in the systematic review and subjected to data extraction. The JBI (Joanna Briggs Institute) Critical Appraisal Checklist for Case-Control Studies was used to perform the risk of bias/quality assessment of the six included studies (Table 3).

**Table 3:** Critical appraisal of included studies (N=6) ^Q1. Were the groups comparable other than the presence of disease in cases or the absence of disease in controls?^ ^Q2. Were cases and controls matched appropriately?^ ^Q3. Were the same criteria used for identification of cases and controls?^ ^Q4. Was exposure measured in a standard, valid and reliable way?^ ^Q5. Was exposure measured in the same way for cases and controls?^ ^Q6. Were confounding factors identified?^ ^Q7. Were strategies to deal with confounding factors stated?^ ^Q8. Were outcomes assessed in a standard, valid and reliable way for cases and controls?^ ^Q9. Was the exposure period of interest long enough to be meaningful?^ ^Q10. Was appropriate statistical analysis used?^

Study (Author(s), Year)	Q1	Q2	Q3	Q4	Q5	Q6	Q7	Q8	Q9	Q10
Balasubramaniam et al., 2013 [35]	Yes	No	Yes	Yes	Yes	Yes	Unclear	Yes	Yes	Yes
Ganesh et al., 2011 [36]	Yes	No	Yes	Yes	Yes	Yes	Yes	Yes	Unclear	Yes
Moirangthem et al., 2024 [37]	Yes	Yes	Yes	Yes	Yes	Yes	Yes	Yes	Yes	Yes
Rao et al., 2002 [38]	Yes	No	Yes	Yes	Yes	Yes	Yes	Yes	Yes	Yes
Dar et al., 2014 [39]	Yes	Yes	Yes	Yes	Yes	Yes	Yes	Yes	Yes	Yes
Joseph et al., 2016 [40]	Yes	Yes	Yes	Yes	Yes	Yes	Yes	Yes	Yes	Yes
(Yes/6) %	100.0	50.0	100.0	100.0	100.0	100.0	83.33	100.0	83.33	100.0

The six selected studies collectively involved a total of 12,360 patients, including 3,425 cases and 8,935 controls, all meeting the predefined inclusion criteria and had data on occupational cancer.

Characteristics of the Included Studies

The key characteristics of the included studies, with reference to the author’s name, year of publication, state, study design, study duration, population type, sample size with case-control distribution, gender, sample size, age, type of cancer, and occupations reported, matching criteria, and outcomes have been reported.

The six studies included in this review were conducted across various states in India, with four originating from Mumbai, Maharashtra, one from Kashmir, and one from Mangalore, Karnataka [35-40]. The average age of the participants ranged from 45.97 to 61.6 years for cases and 44.45 to 59.8 years for controls. Three studies exclusively involved male participants, two included both sexes, and a single study focused solely on female participants.

All included studies employed a hospital-based case-control study design. They investigated five different occupational groups, including pesticide workers, farmers and domestic workers, beedi workers, and agricultural laborers. These studies examined the association between occupational exposures and the risk of different types of cancers, such as lymphoma, oesophageal squamous cell carcinoma (ESCC), lung cancer, cervical carcinoma, buccal mucosa cancer, and gastrointestinal tract cancers. Smoking was consistently identified as a potential confounding factor, and all studies adjusted their findings to account for its influence. The findings of this systematic review are reported in detail in Table 4.

**Table 4:** Characteristics of the studies included in the systematic review (N=6) *Statistically Significant; ^a^ Average age; ^b^ Mean±SD ISCO: International Standard Classification of Occupation; OR: odds ratio; ESCC: esophageal squamous cell carcinoma; χ2: Chi square value, CI: confidence interval; BMC: buccal mucosa cancer; NHL: non Hodgkin's lymphoma

Author(s)	Year	Location in India	Study design	Year	Population type	Case Vs Control	Sex	Case-age Vs Control-age (in years)	Matching	Cancer Reported	Occupation reported	Adjusted Factors	Outcomes
Balasubramaniam et al. [35]	2014	Mumbai	Case-control study	1997-99	Lymphoma patient	390 cancer patients versus 1383 cancer-free patients	Male	46.1^a^ versus 46.4^a^	Unmatched	Non-Hodgkin Lymphoma	Pesticides worker	Age, literacy status, cigarette smokers, and beedi smokers, adjusted for each other	Exposure to pesticides showed a 3-fold excess risk for NHL (OR 3.1*; p<0.01; CI (1.5-6.2))
Ganesh et al. [36]	2011	Mumbai	Case-control study	1997-99	Lung Cancer	408 cancer patients versus 1383 cancer-free patients	Male	56.2^a^ versus 46.5^a^	Unmatched	Lung cancer	Pesticides worker	Age, literacy status, cigarette smokers, and beedi smokers	Exposure to pesticides showed a 2.5-fold enhanced significant risk for lung cancer (OR 2.5; 95%CI (1.2-6.4))
Moirangthem et al. [37]	2024	Mumbai	Case-control study	2010–2022	Buccal mucosa cancer	1969 BMC cases versus 2145 visitors of cancer patients	Males	45.97^a^ versus 44.45^a^	Age (10-year interval) and current residential zone	Buccal mucosa cancer	ISCO-08^#^occupational groups	Tobacco and alcohol consumption	Increased risk of BMC was observed in ‘Craft and Related Trades Workers’ (OR 1.37; 95%CI 1.13–1.65), ‘Plant and Machine Operators and Assemblers’ (OR: 1.26; 95%CI 1.01–1.56), and ‘Elementary Occupations’ (OR:1.33; 95%CI 1.12–1.58).
Rao et al. [38]	2002	Mumbai	Case-control study	1988–1992	Gastrointestinal tract cancer	170 cancer patients versus 2184 cancer-free patients	Male and Female	51.9±11.1^b^ versus 48.1±11.4^ b^	Unmatched	Gastrointestinal tract cancers	Agricultural workers	Five age groups, residence, and habits, including smoking and tobacco chewing, for male participants only	Male agricultural workers had a 50% excess risk (OR=1.5, 95%CI 1.1–2.6) compared to other occupations, whereas labourers and mill/textile workers did not show as high a risk.
Dar et al. [39]	2014	Kashmir	Case-control study	September 2008 to January 2012	ESCC	703 patients (not having a strong association with tobacco or alcohol consumption) versus 1664 controls	Male and Female	61.6±11.1^b^ versus 59.8±11.1^b^	Age, sex, and district of residence	Oesophageal squamous cell carcinoma (ESCC)	Farmers and domestic workers	Age, ethnicity, place of residence, fruit and vegetable intake (logarithmic scale), ever use of alcohol, and cumulative use of hookah, cigarette, and nass (a mixture of tobacco, ash, lime, oil, and flavouring and colouring agents).	Occupation as farming and domestic workers who had long-term and daily close contact with animals was strongly associated with a high risk of ESCC (OR 6.01; 95%CI (2.49 - 14.55).
Joseph et al. [40]	2016	Mangalore	Case-control study	2011-12	Cervical Carcinoma	175 cancer patients versus 176 non-cervix carcinoma controls	Female	55.2±11.5^b^ versus 55.3±12.0^b^	Five-year age group matched control from the same hospital, admitted with a disease not associated with tobacco usage.	Cervix carcinoma	Beedi worker	Tobacco chewing among participants	Exposure rate to tobacco dust following beedi rolling was 63 (26.4%) among cases and 38 (15.9%) among controls (χ2 = 7.846, P = 0.005, OR = 1.893, 95%CI: 1.207–2.971). Beedi rolling in association with the development of cervical cancer was found to be 1.913 (95%CI: 1.215– 3.01), (P = 0.005).

Discussion

This systematic review presents a detailed analysis of occupational cancers in India, shedding light on the significant risks associated with specific professions such as agriculture, domestic work, pesticide spraying, and beedi production. The findings are consistent with international research, including the Nordic Occupational Cancer (NOCCA) project and the Global Burden of Disease (GBD) 2017 comparative risk assessment, which highlights the pervasive role of occupational exposures in cancer development [41,42]. Globally, the NOCCA project reported elevated standardized incidence ratios (SIRs) for cancers in professions such as tobacco handling, chimney sweeping, seafaring, plumbing, and woodwork. The GBD 2017 assessment further identified key carcinogens, including asbestos, silica, solar radiation, and diesel exhaust, as contributors to the global burden of occupational cancers. These findings align with the Indian context, where pesticide workers exhibited a three-fold increased risk of lymphoma (OR 3.1, 95%CI: 1.5-6.2) [43]. Similarly, farmers and domestic workers with prolonged animal contact were found to have a six-fold higher risk of oesophageal squamous cell carcinoma (OR 6.01, 95%CI: 2.49-14.55) [39]. Among beedi workers, exposure to tobacco dust was significantly associated with cervical carcinoma (OR 1.913, 95%CI: 1.215-3.01) [40].

The demographic and methodological diversity among the studies included in this review highlights the complexity of occupational cancer research. All six studies employed hospital-based case-control designs, collectively involving 12,360 participants, with 3,425 cases and 8,935 controls. Sex-specific trends were evident, with three studies exclusively focusing on male participants [35-37], two including both male and female participants [38,39], and one studying only female participants [40]. The studies consistently identified smoking as a confounding factor, necessitating statistical adjustments to isolate the impact of occupational exposures.

Occupational cancers in India exhibit a diverse and complex etiological profile, with various occupations linked to heightened cancer risk. Pesticide application, for instance, was linked to both lymphoma and lung cancer, underscoring the hazardous nature of chemical exposures in agriculture (OR 2.5, 95%CI: 1.2-6.4) [36]. Domestic workers and farmers with routine animal contact demonstrated an elevated risk of oesophageal cancer, emphasizing the need for further research into zoonotic and environmental carcinogens. Beedi workers, exposed to tobacco dust, faced a disproportionately high risk of cervical carcinoma, while buccal mucosa cancer was prevalent among International Standard Classification of Occupations 2008 (ISCO-08) occupational groups such as craft workers and machine operators (OR range: 1.26-1.37) [37]. Additionally, agricultural laborers exhibited a 50% higher risk of gastrointestinal tract cancers compared to other occupations (OR 1.5, 95%CI: 1.1-2.6) [38].

In the context of lung cancer, significantly elevated risks were observed when comparing individuals who were ever employed in specific occupations to those never employed in the same roles. Adjusted for smoking, textile workers (OR 1.99; 95%CI: 1.3-3.6) and cooks (OR 4.48; 95%CI: 1.2-16.9) had markedly increased risks, with similar trends observed among ship and dockyard workers (OR 2.87; 95%CI: 0.8-10.1) and wood workers (OR 2.88; 95%CI: 0.9-9.6) [43]. Additionally, literature demonstrated that sugarcane farm workers have a significantly higher risk of lung cancer compared to non-exposed groups (OR 1.92; 95%CI: 1.08-3.40) [44]. For bladder cancer, elevated risks were noted exclusively among chemical and pharmaceutical plant workers (OR 4.48; 95%CI: 1.2-16.5) [43]. These findings underline the need for public health interventions and protective measures for high-risk occupational groups.

Insights from international studies provide valuable comparative perspectives. The Health and Safety Executive (HSE) 2024 report from Great Britain highlights an alarming rise in occupational cancer incidence, attributed to both confirmed (IARC Group 1) and probable (IARC Group 2A) carcinogens [45]. Asbestos remains the leading occupational carcinogen, responsible for a substantial proportion of lung cancer and mesothelioma cases. Other significant contributors include exposure to silica dust, solar UV radiation, diesel exhaust, and wood dust [46]. These global trends mirror the occupational hazards identified in India, emphasizing the universal nature of these risks.

Addressing the burden of occupational cancer requires multifaceted strategies encompassing policy, research, and healthcare interventions. Strengthened regulations governing pesticide use and industrial safety standards are paramount to minimizing exposure risks. Awareness campaigns targeting high-risk populations, particularly in rural and underserved areas, can further mitigate the impact of occupational hazards. In addition, longitudinal studies are essential to unravel causal pathways and understand the latency periods associated with various occupational cancers. Advanced exposure assessment methodologies, such as biomonitoring and geospatial analytics, can enhance the precision of risk estimation.

Healthcare systems must prioritize robust screening and early detection programs tailored to high-risk occupational groups. For instance, regular health check-ups and cancer screenings for pesticide workers, farmers, and beedi workers can facilitate early diagnosis and improve treatment outcomes. Moreover, post-diagnosis support services, including rehabilitation and counseling, should be made accessible to marginalized populations.

Global collaboration plays a crucial role in addressing occupational cancer. International partnerships can facilitate the exchange of data, research methodologies, and best practices, thereby enhancing the efficacy of prevention and management strategies. Collaborative initiatives, such as the NOCCA project and the GBD comparative risk assessments, underscore the potential of collective efforts in mitigating occupational health risks.

The findings of this review underscore the significant burden of occupational cancers in India, with specific professions and exposures contributing disproportionately to this public health challenge. The parallels with global trends highlight the need for a unified approach to address occupational cancer risks. Through targeted policy interventions, enhanced research endeavors, and improved healthcare systems, the burden of occupational cancer can be effectively reduced, fostering better health outcomes for workers both in India and globally. Future efforts should prioritize interdisciplinary collaboration and innovation to tackle this pressing issue comprehensively.

## Conclusions

This systematic analysis of six case-control studies from diverse regions of India demonstrates a consistent and significant association between occupational exposures and multiple cancer types. Across studies, workers engaged in pesticide application, farming, beedi rolling, and industrial or craft-related occupations exhibited substantially elevated risks of cancers such as non-Hodgkin lymphoma, lung cancer, cervical cancer, oesophageal squamous cell carcinoma, buccal mucosa cancer, and gastrointestinal tract cancers. Exposure to agrochemicals, tobacco dust, and industrial pollutants emerged as recurring risk determinants, even after adjustment for major confounders including age, tobacco and alcohol use, literacy, and other demographic variables. Notably, pesticide exposure showed 2.5-3-fold increased risks for hematological and respiratory cancers, while occupations involving close contact with animals or agricultural environments demonstrated up to a six-fold higher risk of oesophageal cancer.

Similarly, beedi workers and labour-intensive industrial groups faced increased risks of cervical and buccal mucosa cancers, respectively. Agricultural work among males also conferred approximately 50% excess gastrointestinal cancer risk, highlighting the breadth of occupational carcinogenic hazards across sectors. Collectively, the evidence indicates a robust and biologically plausible link between occupational factors and cancer development in the Indian context. These findings underscore the urgent need for strengthened occupational health surveillance, regulation of hazardous exposures, worker education, and targeted preventive strategies within high-risk industries. Further large-scale, prospective studies are warranted to refine exposure assessment and guide policy interventions aimed at reducing the burden of occupational cancers in India.

## References

[1] Hashim D, Boffetta P (2014). Occupational and environmental exposures and cancers in developing countries. Ann Glob Health.

[2] Straif K, Benbrahim-Tallaa L, Baan R (2009). A review of human carcinogens--Part C: metals, arsenic, dusts, and fibres. Lancet Oncol.

[3] Samet JM, Chiu WA, Cogliano V (2020). The IARC monographs: updated procedures for modern and transparent evidence synthesis in cancer hazard identification. J Natl Cancer Inst.

[4] (2026). Carcinogens: different classifications. https://www.prevor.com/en/carcinogens-different-classifications/.

[5] Prüss-Ustün A, Wolf J, Corvalán C, Bos R, Neira M (2016). Preventing disease through healthy environments: Towards an estimate of the environmental burden of disease. Preventing Disease Through Healthy Environments: A Global Assessment of the Burden of Disease From Environmental Risks.

[6] Bray F, Laversanne M, Sung H, Ferlay J, Siegel RL, Soerjomataram I, Jemal A (2024). Global cancer statistics 2022: GLOBOCAN estimates of incidence and mortality worldwide for 36 cancers in 185 countries. CA Cancer J Clin.

[7] Im S, Youn KW, Shin D, Lee MJ, Choi SJ (2015). Review of carcinogenicity of asbestos and proposal of approval standards of an occupational cancer caused by asbestos in Korea. Ann Occup Environ Med.

[8] Manno M, Levy L, Johanson G, Cocco P (2018). Silica, silicosis and lung cancer: what level of exposure is acceptable?. Med Lav.

[9] Speer RM, Zhou X, Volk LB, Liu KJ, Hudson LG (2023). Arsenic and cancer: evidence and mechanisms. Adv Pharmacol.

[10] Riudavets M, Garcia de Herreros M, Besse B, Mezquita L (2022). Radon and lung cancer: current trends and future perspectives. Cancers (Basel).

[11] El Ghissassi F, Baan R, Straif K (2009). A review of human carcinogens--part D: radiation. Lancet Oncol.

[12] Jacobs MM, Massey RI, Tenney H, Harriman E (2014). Reducing the use of carcinogens: the Massachusetts experience. Rev Environ Health.

[13] Burki T (2010). Health experts concerned over India’s asbestos industry. Lancet.

[14] (2025). World Health Organization: Occupational health. https://www.who.int/india/health-topics/occupational-health.

[15] Dar MA, Sharma KK (2019). Burden of cancer in India: GLOBOCAN 2018 estimates incidence, mortality, prevalence and future projections of cancer in India. J Emerg Tech Innov Res.

[16] Jadhav AV, Gawde NC (2019). Current asbestos exposure and future need for palliative care in india. Indian J Palliat Care.

[17] Prajapati SS, Nandi SS, Deshmukh A, Dhatrak SV (2020). Exposure profile of respirable crystalline silica in stone mines in India. J Occup Environ Hyg.

[18] Recommendations of 12th National Conference on Safety in Mines.

[19] (2026). Occupational Safety and Health Administration: Respirable crystalline silica. https://www.osha.gov/laws-regs/regulations/standardnumber/1910/1910.1053.

[20] Misra S, Sussell AL, Wilson SE, Poplin GS (2023). Occupational exposure to respirable crystalline silica among US metal and nonmetal miners, 2000-2019. Am J Ind Med.

[21] (2003). Decree of Minister of Manpower and Immigration no. 211 for 2003 on Safety Levels, Precautions, and Terms to Prevent Detrimental Physical, Chemical, Biological, and Mechanical Hazards and Securing the Work Environment.

[22] Sathishkumar K, Chaturvedi M, Das P, Stephen S, Mathur P (2022). Cancer incidence estimates for 2022 & projection for 2025: result from National Cancer Registry Programme, India. Indian J Med Res.

[23] Hnizdo E, Vallyathan V (2003). Chronic obstructive pulmonary disease due to occupational exposure to silica dust: a review of epidemiological and pathological evidence. Occup Environ Med.

[24] Methner MM, Page EH (2017). Evaluation of Exposure to Crystalline Silica, Welding Fume, and Isocyanates During Water Heater Manufacturing: Methner, Mark M.;Page, Elena H.; National Institute for Occupational Safety and Health, Published. Evaluation of Exposure to Crystalline Silica, Welding Fume, and Isocyanates During Water Heater Manufacturing: Health Hazard Evaluation Report 2015-0076-3282.

[25] Picciotto S, Neophytou AM, Brown DM, Checkoway H, Eisen EA, Costello S (2018). Occupational silica exposure and mortality from lung cancer and nonmalignant respiratory disease: G-estimation of structural nested accelerated failure time models. Environ Epidemiol.

[26] Stayner L, Welch LS, Lemen R (2013). The worldwide pandemic of asbestos-related diseases. Annu Rev Public Health.

[27] Kouokam JC, Speer RM, Meaza I, Toyoda JH, Lu H, Wise JP Sr (2024). Transcriptomic analysis reveals particulate hexavalent chromium regulates key inflammatory pathways in human lung fibroblasts as a possible mechanism of carcinogenesis. Toxicol Appl Pharmacol.

[28] Hesterberg TW, Long CM, Bunn WB, Lapin CA, McClellan RO, Valberg PA (2012). Health effects research and regulation of diesel exhaust: an historical overview focused on lung cancer risk. Inhal Toxicol.

[29] Lan Q, Zhang L, Li G (2004). Hematotoxicity in workers exposed to low levels of benzene. Science.

[30] Shankar A, Dubey A, Saini D (2019). Environmental and occupational determinants of lung cancer. Transl Lung Cancer Res.

[31] Singh Z, Chadha P (2016). Textile industry and occupational cancer. J Occup Med Toxicol.

[32] Zhang Y, Mi M, Zhu N (2023). Global burden of tracheal, bronchus, and lung cancer attributable to occupational carcinogens in 204 countries and territories, from 1990 to 2019: results from the global burden of disease study 2019. Ann Med.

[33] Khetan M, Babu BV (2025). Silicosis prevalence and related issues in India: a scoping review. J Occup Med Toxicol.

[34] Page MJ, McKenzie JE, Bossuyt PM (2021). The PRISMA 2020 statement: an updated guideline for reporting systematic reviews. BMJ.

[35] Balasubramaniam G, Saoba S, Sarade M, Pinjare S (2013). Case-control study of risk factors for non-Hodgkin lymphoma in Mumbai, India. Asian Pac J Cancer Prev.

[36] Ganesh B, Sushama S, Monika S, Suvarna P (2011). A case-control study of risk factors for lung cancer in Mumbai, India. Asian Pac J Cancer Prev.

[37] Moirangthem R, Hosseini B, Patil A (2024). Occupations and the risk of buccal mucosa cancer in Indian men: a multi-centre case-control study. Cancer Epidemiol.

[38] Rao DN, Ganesh B, Dinshaw KA, Mohandas KM (2002). A case-control study of stomach cancer in Mumbai, India. Int J Cancer.

[39] Dar NA, Islami F, Bhat GA (2014). Contact with animals and risk of oesophageal squamous cell carcinoma: outcome of a case-control study from Kashmir, a high-risk region. Occup Environ Med.

[40] Joseph N, Nelliyanil M, Supriya K (2016). Association between occupational history of exposure to tobacco dust and risk of carcinoma cervix: a case-control study. Indian J Cancer.

[41] Pukkala E, Martinsen JI, Lynge E (2009). Occupation and cancer - follow-up of 15 million people in five Nordic countries. Acta Oncol.

[42] (2018). Global, regional, and national comparative risk assessment of 84 behavioural, environmental and occupational, and metabolic risks or clusters of risks for 195 countries and territories, 1990-2017: a systematic analysis for the Global Burden of Disease Study 2017. Lancet.

[43] Notani PN, Shah P, Jayant K, Balakrishnan V (1993). Occupation and cancers of the lung and bladder: a case-control study in Bombay. Int J Epidemiol.

[44] Amre DK, Infante-Rivard C, Dufresne A, Durgawale PM, Ernst P (1999). Case-control study of lung cancer among sugar cane farmers in India. Occup Environ Med.

[45] (2025). Health and Safety Executive: Health and safety statistics. https://www.hse.gov.uk/statistics/index.htm.

[46] Collatuzzo G, Turati F, Malvezzi M, Negri E, La Vecchia C, Boffetta P (2023). Attributable fraction of cancer related to occupational exposure in Italy. Cancers (Basel).

